# Context-Aware Gossip-Based Protocol for Internet of Things Applications

**DOI:** 10.3390/s18072233

**Published:** 2018-07-11

**Authors:** Lina Altoaimy, Arwa Alromih, Shiroq Al-Megren, Ghada Al-Hudhud, Heba Kurdi, Kamal Youcef-Toumi

**Affiliations:** 1 Information Technology Department, King Saud University, Riyadh 12371, Saudi Arabia; laltoaimy@ksu.edu.sa (L.A.); salmegren@ksu.edu.sa (S.A.-M.); galhudhud@ksu.edu.sa (G.A.-H.); 2 Computer Science Department, King Saud University, Riyadh 12371, Saudi Arabia; aalromih@ksu.edu.sa; 3 Mechanical Engineering Department, Massachusetts Institute of Technology (MIT), Cambridge, MA 02139, USA; youcef@mit.edu

**Keywords:** Internet of Things (IoT), wireless sensor network (WSN), gossiping protocol, context-aware, content-aware, routing protocol

## Abstract

This paper proposes a gossip-based protocol that utilises a multi-factor weighting function (MFWF) that takes several parameters into account: residual energy, Chebyshev distances to neighbouring nodes and the sink node, node density, and message priority. The effects of these parameters were examined to guide the customization of the weight function to effectively disseminate data to three types of IoT applications: critical, bandwidth-intensive, and energy-efficient applications. The performances of the three resulting MFWFs were assessed in comparison with the performances of the traditional gossiping protocol and the Fair Efficient Location-based Gossiping (FELGossiping) protocol in terms of end-to-end delay, network lifetime, rebroadcast nodes, and saved rebroadcasts. The experimental results demonstrated the proposed protocol’s ability to achieve a much shorter delay for critical IoT applications. For bandwidth-intensive IoT application, the proposed protocol was able to achieve a smaller percentage of rebroadcast nodes and an increased percentage of saved rebroadcasts, i.e., better bandwidth utilisation. The adapted MFWF for energy-efficient IoT application was able to improve the network lifetime compared to that of gossiping and FELGossiping. These results demonstrate the high level of flexibility of the proposed protocol with respect to network context and message priority.

## 1. Introduction

Interconnectivity between dissimilar devices to form a pervasive network of services is possible among a broad variety of electronic devices due to the miniaturisation of these devices and reductions in their cost [1]. Such electronic devices are made smart by endowing physical things with local intelligence, thereby bridging the gulf between the physical world and the world of information [2]. The network connecting these smart devices, i.e., intelligent things, has been termed the Internet of Things (IoT) [3]. IoT technology is driving the third wave of the information technology (IT) revolution, which is striving towards an era of ubiquity, heterogeneity, and connectivity [4]. This revolution is occurring in concert with the popularisation of wireless and sensor technologies enabled by wireless sensor networks (WSNs) [5,6]. The IoT is a communicating-actuating network of devices that augment digital information with information about the physical world, i.e., the environment around us [7]. In other words, devices in an IoT network dynamically cooperate to fulfil an IoT application’s goal.

An IoT network is characterised by its ability to offer comprehensive perception, reliable transmission, and intelligent processing [1]. Identification technology and sensors are utilised in an IoT network to enable people to interact with the real world remotely via a variety of wired and wireless technologies for transmission and communication among machines (i.e., machine-to-machine, or M2M, communication) and with the users of IoT applications. Within the context of the IoT, WSNs permit the utilisation of different network topologies and multi-hop communication to enable the monitoring of physical and environmental conditions via spatially distributed devices. The instantaneous and intelligent processing of the collected data is facilitated by intelligent computing technologies, e.g., cloud computing, and the results are communicated to humans or other devices. Human-to-device communication is implemented in IoT applications to provide reliable and robust interaction. Device-to-device interactions are also made possible in IoT applications through the utilisation of intelligent devices that are able to identify a problem, communicate effectively, and resolve the issue without human intervention [8].

The capabilities of IoT applications are varied and depend on the types of services being offered by said applications. For instance, IoT applications for smart agriculture (e.g., [9]) are currently being used for the clean and sustainable growing of food. An application of this sort requires the deployment of a large number of sensors to detect environmental and ecological factors to support the remote control of actuators based on those readings. As another example, an IoT application intended for electronic health (e-health) monitoring (e.g., [10]) collects and analyses data from an ad hoc network of several devices that are placed on a patient’s body to provide end users with feedback on health-related habits and advice. The performance of IoT applications is further impacted by the capabilities of the devices being utilised and of the network itself. This is because most IoT devices are resource-challenged devices, with limited memory, processing capabilities, electrical energy supplies, and other abilities [11].

Often, an IoT application requires additional computational power to process the data collected by the sensors. This requires the sensors to forward their data to a control station (i.e., sink) for further operations. This is accomplished through efficient routing protocols, such as gossiping protocols, which can, in turn, impact the performance of the IoT network and application. Gossiping is a traditional communication mechanism that mimics the behaviour of common dissemination phenomena, such rumours or epidemics. In a traditional gossiping protocol, a message is generated by a source and is propagated to the sink node with the active participation of most nodes in the network to ensure that the message is received at the sink [12]. A sender node (beginning with the source node) transmits the message to a single neighbour chosen at random. The selected node then chooses another random neighbour to which to forward the packet. This process is repeated until the message is received at the destination, i.e., the sink node [13]. It is evident from this simple procedure that message redundancy may be an issue, as the same message can be received more than once. Energy is also wasted due to the participation of most of the nodes in the network in the dissemination of a message, directly impacting the network’s lifetime. These challenges can potentially be remedied through the utilisation of election scores for optimisation to limit the number of participating nodes.

**Figure 1:**
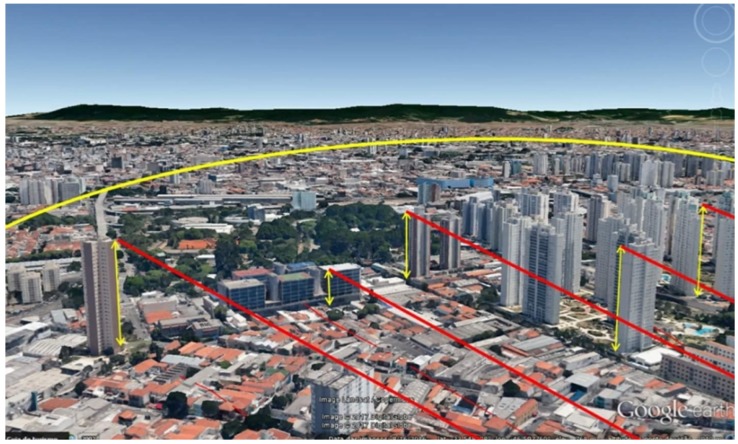
Smart city project. Reproduced with permission from Dalton Oliveria [17], SmartyTempy-IoT smartyWiot for Smart Cities [18]; published by Wardston Consulting, 2018.

IoT applications can provide access to comprehensive intelligent services and information [1,14]. This can be achieved by the utilisation of the underlying sensor networks. Therefore, the performance of such application will depend on the communication protocol that facilities the data dissemination among sensors in the network. However, the problem with designing such protocol is tightly related to the underlying sensor network, where sensors have limited resources and capabilities, particularly in terms of power consumption, storage capacity, and bandwidth. In addition, the emerging use of fog computing in IoT applications has raised the need for an efficient multi-hop communication mechanism [15]. In fog computing, sensed data would be sent to the network’s edge device (i.e., sink node), rather than a gateway device. This should reduce network traffic and enhance the performance of IoT applications [16]. In smart city environments, communication has been introduced as a customised solution to resolve the issue with Internet connectivity due to obstacles that impede transmission [17,18]. In a recent smart city project, three different types of stations are considered: (1) sender, (2) sender/receiver, and (3) receiver. Information is gathered from the level of the street (type 1 stations) to the top of the building (type 2 stations) and then to another building (type 3 station). Therefore, the information is passed through the stations as follows: (1) > (2) > (3). The reason for using type 2 stations is because type 1 stations cannot directly communicate to type 3 station due to buildings and obstacles that exist in the way. In addition, stations 1 and 2 do not have Internet connection, while stations 3 do. This solution is illustrated in Figure 1.

In this paper, a communication mechanism based on a gossiping protocol is proposed. The new protocol utilises knowledge regarding the network context and message priority. The protocol is made context-aware by its ability to gather information about its environment, i.e., neighbors and sink, to adapt its procedure and guide its response accordingly. The main contributions of this paper are threefold. First, a new protocol is proposed that uses a multi-factor weight function (MFWF) to allow the sensors in an IoT network to effectively communicate and propagate messages. This weight function takes several parameters into account: residual energy, Chebyshev distances to neighbouring nodes and to the sink node, node density, and message priority. Second, a simulation model is presented that was built to examine the performance of the proposed protocol in terms of the end-to-end delay, rebroadcast nodes, saved rebroadcasts, and network lifetime by examining each parameter individually. The findings show that the choice among parameters or certain combinations of parameters depends on the application requirements. Third, this paper reports further simulations that were conducted to examine the impact of parameter variability on three categories of applications within the IoT context: critical, bandwidth-intensive, and energy-efficient applications. In these simulations, the performance of the proposed protocol was evaluated in comparison with the original gossiping protocol and the Fair Efficient Location-based Gossiping (FELGossiping) protocol [19]. The experimental results show that the proposed protocol better satisfies the requirements of each analysed type of IoT applications: it reduces the end-to-end delay for critical applications, achieves fewer rebroadcast nodes and an increased percentage of saved broadcasts for bandwidth-intensive applications, and improves the network lifetime for energy-efficient applications.

The remainder of this paper is organised as follows. Section 2 reviews variants of the gossiping protocols for WSNs. Next, the motivating scenario underlying this work is presented in Section 3. The system design is introduced in Section 4, detailing the weight function, the parameters considered, and the proposed variant of the gossiping protocol. In Section 5, the experimental configuration used for conducting the simulations is described. This section also presents and discusses the experimental results. Section 6 assesses the performance of the proposed gossiping protocol in comparison with well-established benchmarks and reports the results. Finally, Section 7 summarises and concludes the paper and briefly discusses future work.

## 2. Related Work

Various characteristics of gossiping protocols have been investigated, including their handling of bounded and small-sized messages, low-frequency interactions between nodes, random peer selection, and the unreliable interaction assumption [20]. The power of a gossiping protocol lies in its symmetric simplicity, meaning that each node runs identical algorithms. A gossiping protocol will also operate correctly and adequately on a variety of topologies; thus, such protocols can arguably be considered topologically independent. Well-designed gossiping protocols can also eliminate the risk of disruptive load surges due to their production of bounded, almost negligible, loads. Despite these advantages, gossiping protocols also suffer from several limitations due to their bounded message sizes and low periodic message exchange rates. These limitations result in a distributed system with a severely limited information carrying capacity. The rest of this section reviews several research efforts that have focused on proposing modifications to the original gossiping protocol to address its limitations.

The Location-based Gossiping (LGossiping) protocol was developed as an improved gossiping-based method of message distribution that is based on the locations of all nodes in the network [21]. This means that nodes reliably forward messages to known neighbours using the Global Positioning System (GPS). According to a comparative performance analysis with the original gossiping protocol as the benchmark, LGossiping, to some extent, solves the delay problem of the original protocol. Nevertheless, there are many instances in which the data fail to reach the sink. In addition, the network size was fixed to 100 nodes throughout the experiments. An improved version of the LGossiping protocol, Energy Location-based Gossiping (ELGossiping), bases its selection of the next node on its distance to the sink [22]. Therefore, when an event is detected by a node, the next hop is determined by selecting the neighbouring node with the maximum energy and the minimum distance to the sink. The performance of ELGossiping in terms of the number of live nodes, the energy consumption, and the packet loss was comparatively assessed against the performances of Gossiping and LGossiping. The findings showed that ELGossiping outperforms the other two protocols, with an increased network lifetime, a reduced number of hops and lower energy consumption. However, the authors did not mention how the distance to the main station is measured or determined.

A chance-based gossiping protocol, CHGossiping, was proposed to overcome the delay problems of the LGossiping algorithm and to reduce the number of non-input events at the sink [23]. Similar to ELGossiping [22], this routing algorithm considers both a node’s energy and its distance to the sink. In CHGossiping, the next-hop node is randomly selected with a probability based on the remaining energy of a node and its distance to the sink. A node’s chance for selection is increased when it is closer to the sink and contains more residual energy. This approach decreases the delay and consumes energy more efficiently. The performance of CHGossiping was comparatively assessed against the performances of the original gossiping protocol and LGossiping [21] with regard to the network lifetime, energy consumption, and packet loss. The findings showed that CHGossiping performs better than the other two routing protocols; it proved to be more reliable and energy efficient, with an increased network lifetime. Although the chance parameter is calculated using both the node’s energy and distance to sink, it is still sent in the node greeting packet along with the other parameters. This would cause some network overhead which was not considered in the design of the CHGossiping protocol.

The Fair Efficient Location-based Gossiping (FELGossiping) protocol was later proposed to control the energy consumption of the nodes in a WSN during communication and thus to increase the network’s lifetime [19]. The selection of the next node in FELGossiping is based on its residual energy and distance to the sink. In FELGossiping, when a node detects an event and wants to send information, it determines its next hop based on its neighbouring nodes’ residual energy levels and hop counts. This procedure continues until the message reaches the sink. Thus, it effectively increases the energy available to the network and maximises its lifetime. The performance of the proposed protocol was compared with the performances of three other algorithms, namely, the original gossiping protocol, LGossiping [21], and ELGossiping [22], in terms of the packet loss, number of live nodes, energy consumption, and delay. FELGossiping outperformed the other protocols by achieving a high packet delivery ratio, reduced delays, a fair energy balance among nodes, and an increased node lifetime. Nevertheless, FELGossiping uses hop counts for distance measurement; however, it is very loosely correlated with the distances as it represents the number of intermediate nodes in which data pass between the source and destination nodes.

Fuzzy-Gossip is a modified gossiping protocol that uses fuzzy logic to reduce the energy waste caused by recirculation redundancies, thus improving the network lifetime [24]. The proposed protocol determines the next node from among a set of candidate nodes by favouring nodes with the highest remaining energy levels and the shortest distances to the sink. This is made possible through the use of fuzzy logic to determine a selection cost for each candidate node based on a series of five membership functions. The effectiveness of Fuzzy-Gossip in comparison with three other protocols, i.e., the original gossiping protocol, LGossiping [21], and ELGossiping [22], was demonstrated through simulations. Compared with these protocols, Fuzzy-Gossip was found to reduce message routing redundancy and balance energy consumption more effectively while also maximising the network lifetime. However, this protocol is arguably unscalable because the computational overhead due to the processing of the fuzzified values can lead to high energy consumption.

A probabilistic approach is adopted in a new variant of the gossiping protocol, Gossiping-PS, which introduces new mechanisms for the selection of the next node based on a probability function [25]. The motivation for this proposal was to reduce the routing delay and improve the network lifetime. The probability function is inspired by the ant colony function [26] and determines the node to be selected based on two factors: the residual energy of the node and its visibility [27]. The node with the highest probability, i.e., a node with a high level of energy and a close distance to the sink, will thus be selected as the next hop for routing. The performance of Gossiping-PS was compared with that of the simple gossiping protocol in terms of the response time, network lifetime, and residual energy. The findings showed that the Gossiping-PS protocol reduces the response time and prolongs the network lifetime in comparison with those achieved with the original gossiping protocol. Nonetheless, the probability function that combines both the residual energy of the node and its visibility was not mentioned in the study.

An energy-efficient variant of the gossiping protocol was proposed based on fuzzy logic and Chebyshev distances [28]. In this approach, a distance-based scheme is adopted to determine the path of a message from its source to its destination by favouring nodes with the most residual energy and the shortest distances to the sink. The performance of this new routing algorithm was compared against those of the traditional flooding and gossiping protocols in terms of the energy consumption, hop count, and network lifetime. The results showed that the new protocol enhances the performance of a WSN and increases its lifespan. Nevertheless, despite the use of the resource-saving Chebyshev distance computation, the fuzzy logic calculations consume considerable computational resources—resources that often need to be preserved to maximise a WSN’s lifetime.

Another gossiping protocol variant, Fitness-Gossiping (FGP), uses a fitness function to select the optimal next node [29]. This is done to decrease the energy consumption of the simple gossiping protocol and thus increase the network lifetime. The fitness function considers the remaining energy, next-node distance, and sink distance in order to select the node with the highest fitness to which to pass along the packet. The proposed protocol was compared against the simple gossiping protocol in terms of the delay, energy consumption, and number of live nodes. The results confirmed that FGP outperforms the simple gossiping protocol by balancing the power consumption more effectively, thereby enhancing the network lifetime. However, the authors compared their work with the traditional gossiping protocols and did not consider other protocols.

Similar to the previously mentioned gossiping protocols, DGossip bases its selection of the next node on its distance to the sink and residual energy [30]. The value used for node selection is computed by means of a factor function that considers each node’s energy and distance to the sink as well as the initial energy and the total distance from the source to the destination. The performance of DGossip was compared against that of the original gossiping protocol to determine its effectiveness in terms of energy, latency, and the number of live nodes. The results showed an improvement in energy dissipation and efficiency when utilising the DGossip protocol, which, in turn, increases the network lifetime and reduces latency. Similar to FGP, the authors did not consider other types of protocols in the evaluation.

Table 1 summarises the reviewed literature on gossiping protocol variants. For package dissemination, the majority of these works adopt various computations that utilise the distance to the sink and/or the residual energy as the primary parameter(s) for determining the next node for selection [22,23,24,25,28,30]. Other parameters examined include the distance to each neighbour node [29], the locations of the nodes [21], and the hop count to the sink [19]. These modified gossiping protocols have typically been evaluated in comparison with the conventional gossiping protocol or other modified gossiping protocols using various metrics. The energy consumption has been most commonly utilised to assess the performance of a proposed protocols against the performances of other protocols in a fixed-size network (100 nodes) [19,21,22,23,24,25,28,29,30], followed by the hop count [22,23,28,29,30], the number of live nodes [19,24,28,29,30], the packet loss [19,21,22,23], and the network lifetime [22,23,25]. The results of these works reflect the positive impact of utilising adjusted parameters and computations to enhance the performance of the original gossiping protocol.

In the context of IoT applications and networks, this paper aims to expand on previous works that have addressed the issues of energy consumption and the distances to the sink and the nearest neighbour in gossiping protocols by also considering the node density (number of one-hop neighbours) and message priority. The impact of each of the five parameters is closely examined for various types of IoT applications to determine the ideal parameter balance that can satisfy the needs of each type of  application.

## 3. Motivating Scenario

This section proposes an example scenario of an IoT system, a smart domiciliary care system, with various underlying requirements and an objective that is inspired by the IEEE’s repository of IoT scenarios [31]. This scenario highlights the need for adjustable and flexible routing protocols that can adapt to changing contexts and message priority. This scenario is used throughout the remainder of this paper to identify and exemplify the examined parameters.

Suppose that Norah works for a domiciliary care facility. She drives every day to the homes of the elderly and the infirm to provide domiciliary care, following the same route each day. In Norah’s city, the municipality has recently implemented a smart domiciliary care system, so that she can now adjust her route in order to reach those most in need first. Each of the homes is equipped with motion detectors and several sensors installed on light switches and water taps, and on-line appointments are offered via videoconferencing for less able residents. Using the information received from these sensors, Norah is able to be notified of any irregularities or emergencies. For instance, if Mr. Ahmed’s water taps were switched on more than average to use the toilet during the night, Norah can now know to book an appointment with Mr. Ahmed’s urologist. More critically, if no motion was sensed in the morning for an early riser such as Mrs. Fatimah, then that could mean that something is very wrong. Norah can therefore urgently change her route to reach Mrs. Fatimah as soon as possible. Such a system can offer a level of security and safety to the infirm (e.g., Mr. Ahmed and Mrs. Fatimah) while also supporting the city in its efforts towards better domiciliary care [32].

## 4. System Design

The main objective of any IoT application is to allow its end users to access services and information that are specific to their time, environment, and location. Therefore, devices and sensors must be aware of their surroundings as well as the main objective of the underlying application. For the purpose of facilitating data dissemination in an IoT environment, this paper proposes a communication mechanism, based on the conventional gossiping protocol, that utilises both the content of the distributed messages and the network context. Designing an efficient mechanism for communication among sensors in an IoT application involves several challenges related to the following concerns:

- Power consumption
- Storage capacity
- Bandwidth limitations

These challenges are addressed in the proposed protocol by allowing the sensors used in an IoT application to communicate effectively using a multi-factor weighting function (MFWF). The proposed content- and context-aware protocol can be simply described as follows:

1. When a source node has new data to send to the sink node, it will first communicate with its neighbouring nodes to gather information related to their energy levels and physical locations.
2. Once the node has gathered the neighbouring nodes’ information, it will assign a weight value to each neighbouring node. The weight value (*w*) is calculated for each neighbouring node as  follows:
  (1) $$w=\sum_{i=1}^{n}f_{i}\cdot p_{i}$$
  where *n* is the total number of parameters used in the function and $f_{i}$ is the weighting factor for parameter $p_{i}$. The value of the weighting factor can be set to either 0 or 1. If it is set to 1, then the corresponding parameter is considered in the calculation; otherwise, it is not.
3. The next node to be selected will depend on the weight value computed. The node with the highest weight value is selected, and the message is forwarded. If multiple neighbouring nodes have the same highest weight value, then the source node will randomly select one node from among them.

This procedure will continue at every selected node, i.e., sender node, until the message is received by the sink node.

The main objective of designing the MFWF as shown in Equation (1) is to allow the impact of each parameter on the efficiency of the underlying IoT application to be investigated individually. Moreover, the MFWF is scalable, as new parameters can be added in the future to further improve efficiency, thus ensuring the continued growth of the proposed protocol to maximise its robustness and flexibility. The detailed procedure is shown in Algorithm 1. The weight function includes the following parameters: residual energy, next-node distance, sink distance, node density, and message priority. The rest of this section describes each of the parameters considered in the MFWF.
**Algorithm 1** The algorithm for the proposed protocol1:E←0.5                                         ▹ Initial energy2:N←TotalNumberOfNodes3:X←SourceNodeXcoordinate4:Y←SourceNodeYcoordinate5:msg←MessagePriority                          ▹ zero if low and one if high6:SelectionList←empty7:neighbours[]←ListOfNeighbours8:$\mathrm{sX}\leftarrow sink^{\prime }sXcoordinate$9:$\mathrm{sY}\leftarrow sink^{\prime }sYcoordinate$10:sToSinkDistance=max(|X−sX|,|Y−sY|)11:**while**Energy>0**do**12:    **if** data sensed ‖ packet received **then**13:        **for all** node *n* : neighbours **do**14:           $\mathrm{nEnergy}\leftarrow n^{\prime }sEnergy$15:           $\mathrm{nX}\leftarrow n^{\prime }sXcoordinate$16:           $\mathrm{nY}\leftarrow n^{\prime }sYcoordinate$17:           $\mathrm{localDensity}\leftarrow n^{\prime }sNumberOfNeighbours$18:           neighbourDistance=max(|X−nX|,|Y−nY|)19:           nToSinkDistance=max(|nX−sX|,|nY−sY|)20:           Energy=nEnergy/E21:           NeighbourDist=1/neighbourDistance22:           sinkDist=1−(nToSinkDistance/sToSinkDistance)23:           nDensity=localDensity/N24:           w=(f1·Energy)+(f2·NeighbourDist)+(f3·sinkDist)+(f4·nDensity)+(f5·msg)25:           add w to *SelectionList*26:        **end for**27:        SortDescending *(SelectionList)*28:        Send data to the first node in *SelectionList*29:    **end if**30:**end while**


### 4.1. Energy

This parameter represents the remaining energy (residual energy, RE) of a neighbouring node. Therefore, when a source node has data to send, it requests its neighbours to send their residual energy values. The energy parameter for a neighbouring node is based on Equation (2):

(2) $$Energy=\frac{RE}{E}$$

where *E* is the node’s initial energy.

### 4.2. Next-Node Distance

This parameter represents the Chebyshev distance between the source node and its neighbouring node. The use of the Chebyshev distance instead of the Euclidean distance is inspired by Dutta et al. [28]. The Chebyshev distance is defined as the maximum difference between two vectors in any coordinate. In a two-dimensional space, the Chebyshev distance between two points *p* and *q* with Cartesian coordinates ($x_{p},y_{p}$) and ($x_{q},y_{q}$) can thus be expressed as $D_{Chebyshev}=max(|x_{p}-x_{q}\left|,\right|y_{p}-y_{q}\left|\right)$.

An assumption is made that the coordinates of all neighbouring nodes are known and distributed throughout the network during the set-up phase. Therefore, the distance can be calculated directly according to Equation (3):

(3) $$NeighbourDist=\frac{1}{max(|X-nX|,|Y-nY|)}$$

where (X,Y) denotes the source node’s coordinates and (nX,nY) denotes the neighbouring node’s coordinates.

### 4.3. Sink Distance

This parameter represents the Chebyshev distance between the neighbouring node and the sink node. The sink distance parameter for each neighbouring node is calculated based on Equations (4)–(6):

(4) sToSinkDistance=max(|X−sX|,|Y−sY|)

(5) nToSinkDistance=max(|nX−sX|,|nY−sY|)

(6) $$sinkDist=1-\frac{nToSinkDistance}{sToSinkDistance}$$

where (sX,xY) denotes the sink node’s coordinates, sToSinkDistance is the Chebyshev distance between the source node and the sink node and nToSinkDistance is the Chebyshev distance between the neighbouring node and the sink node.

### 4.4. Node Density

This parameter represents the fraction of nodes that are considered one-hop neighbours of the neighbouring node. In our proposed protocol, nodes that have a larger number of neighbours are favoured to ensure reliable message delivery. Once the total number of neighbours is determined, the parameter can be calculated according to Equation (7):

(7) $$nDensity=\frac{localDensity}{N}$$

where localDensity is the total number of neighbours of the neighbouring node and *N* is the total number of nodes in the network.

### 4.5. Message Priority

In the IoT context, different types of messages with varying levels of criticality are propagated through a network. For instance, in this article’s motivating scenario (see Section 3), a message indicating an emergency, such as a lack of motion from Mrs. Fatimah, should be given high priority to indicate the need for a fast delivery protocol to ensure that Norah can adjust her route immediately to reach Mrs. Fatimah. In the proposed protocol, messages are classified into two levels of priority: high and low. Setting the message priority will be done through the application itself. Therefore, for high priority messages, this parameter is set to 1; while for low priority messages, this parameter is set to 0. This is shown in Equation (8):

(8) $$msg=\left\{\begin{array}{cc} 0 & low\\ 1 & high \end{array}\right.$$

## 5. Simulations and Parameter Effects

This section describes the simulation environment and its parameters. The evaluation metrics utilised in the simulations to assess the performance of the proposed protocol are also described. The final section presents and discusses the findings from the simulations.

### 5.1. Simulation Environment

A simulator was developed to simulate the performance of the proposed protocol. The program was developed using Java RMI. Parameters similar to those of FELGossiping [19] were used in the simulations. The nodes were randomly deployed in an 80 m × 60 m area with one sink node located at the centre of the area at (40, 30). The locations of the rest of the nodes were randomised. The transmission limit for sensing was set to 20 m. All sensor nodes shared the same initial energy of 0.5 joules. The simulation parameters are summarised in Table 2.

The performance of the proposed protocol was assessed based on a series of evaluation metrics that are relevant within the context of WSNs and the IoT. The evaluation metrics were as follows:

1. *End-to-end delay*: This metric refers to the average time difference between the time at which the message was sent, i.e., the sending time, and the time at which the message was successfully received by the sink node, i.e., the receiving time.
2. *Rebroadcast nodes*: This measure represents the percentage of nodes that have received and rebroadcast the message.
3. *Saved rebroadcasts (SRB)*: This metric is related to the above metric and is used to compute the percentage of bandwidth utilisation. The measure is defined in Equation (9):
  (9) SRB=(v−n)/v
  where *v* is the total number of nodes that have received the message and *n* is the total number of nodes that have retransmitted the message [33].
4. *Network lifetime*: This measure refers to the time from the start of the network simulation up until the death of the last node in the network. This measure represents the number of rounds until the death of the last node in the network [34].

The evaluation framework considers the scalability of the proposed protocol as the number of nodes is increased from 50 to 250 nodes in a fixed-area network. Each simulation was executed ten times with a confidence interval of 95%, and the results were averaged and presented as line graphs, with the number of nodes plotted on the *x*-axis and the performance measure plotted on a linear scale on the *y*-axis.

### 5.2. Simulation Results

The impact of each of the parameters considered in the weight function (see Algorithm 1) on each of the performance measures is presented in this subsection. The effect of each parameter in the proposed MFWF (Equation (1)) was measured by setting that parameter’s weighting factor (*f*) to 1 and the other weighting factors to zero. Table 3 summarises the mapping of the controlled factors to the parameters and their associated equations. The analysis was conducted separately for each of the four evaluation metrics: end-to-end delay, rebroadcast nodes, saved rebroadcasts, and network lifetime. A detailed analysis of the results is presented for each of the four metrics, describing the impact of the five parameters separately on the metric as the size of the WSN network increases from 50 to 250 nodes.

#### 5.2.1. End-to-End Delay

**Figure 2:**
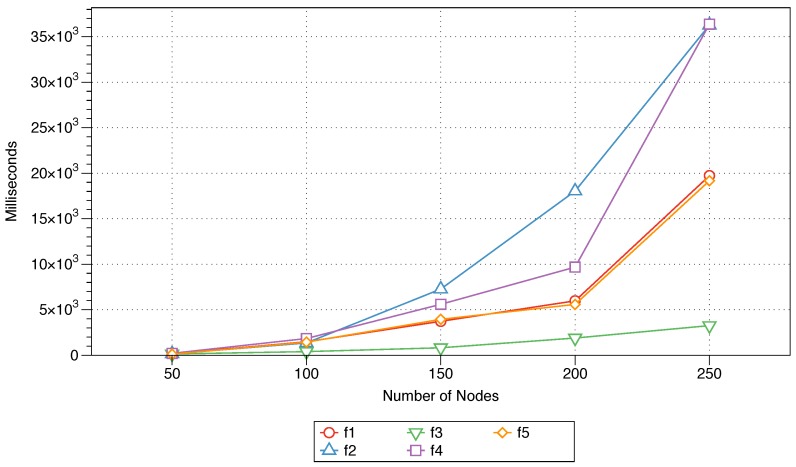
The impact of parameter activation on the average end-to-end delay as the number of nodes increases from 50 to 250 nodes.

The end-to-end delay, as previously defined, is the average time difference between the time the message was sent and the time the message was received by the sink node. In Figure 2, this time is plotted against the number of nodes in a fixed-area network for each of the five parameters considered in the MFWF.

The average end-to-end delay linearly increases as the number of nodes in the network increases for each of the MFWF parameters. However, the sink distance parameter (f3) results in the lowest delay as the number of nodes increases. The variations in this parameter between different numbers of nodes are also much more subtle than those for the other parameters. These findings, as anticipated, highlight the importance of the sink location to the end-to-end delay; that is, the closer the sink node is to a source/neighbouring node, the faster the delivery. The effects of the residual energy (f1) and message priority (f5) on the end-to-end delay are similar and shorter than those of the next-node distance (f2) and node density (f4). Of the five controlled factors, the one associated with the next-node distance parameter (f2) results in the the longest delay and the largest variation as the number of nodes in the network increases.

#### 5.2.2. Rebroadcast Nodes

**Figure 3:**
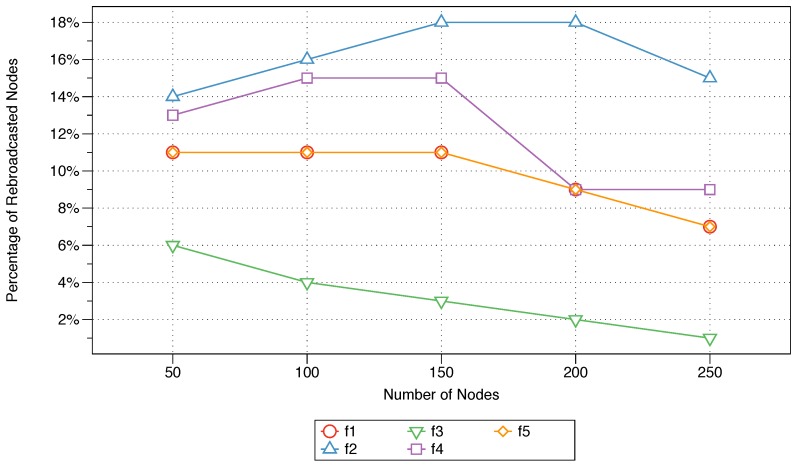
The impact of parameter activation on the average percentage of rebroadcast nodes as the number of nodes increases from 50 to 250 nodes.

The percentage of rebroadcast nodes refers to the percentage of the nodes that have received and rebroadcast the message. To calculate this measure, the total number of nodes that have rebroadcast message is recorded; then, the percentage of rebroadcast nodes is computed relative to the total number of nodes in the network. The percentages were plotted as the number of nodes in the network was increased from 50 to 250 nodes. The effect of parameter activation in the proposed protocol for each of the considered measures is shown in Figure 3.

For the majority of the controlled parameters, the percentage of rebroadcast nodes appears to increase as the number of nodes in the network increases. This is to be expected, since the more nodes there are in a network, the lower the percentage of utilisation given similar parameters for evaluation. However, the influences of the different factors on the rebroadcast node percentage are evident in Figure 3, with values ranging from as little as 1% up to 18%. Of the five parameters, the sink distance (f3) parameter results in the lowest percentages of utilisation across the different numbers of nodes in the network. The impact of the next-node distance (f2) shows the most variability, with the percentage of rebroadcast nodes increasing with the total number of nodes up to a 150-node network and then decreasing as more nodes are introduced into the network. As previously seen in Figure 2, the impacts of the residual energy (f1) and message priority (f5) on the percentage of rebroadcast nodes are similar. The effect of the node density (f4) on this evaluation metric is the second worst, with the percentage only slightly rising as the number of nodes increases from 50 to 100 but dropping by almost 6% as the number of nodes further increases to 200 and 250.

#### 5.2.3. Saved Rebroadcasts

**Figure 4:**
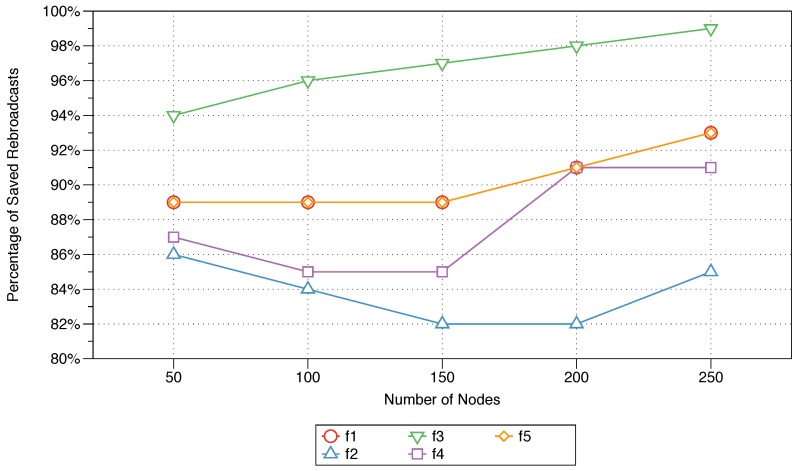
The impact of parameter activation on the average percentage of saved rebroadcasts as the number of nodes increases from 50 to 250 nodes.

The saved rebroadcasts performance measure is used to determine the bandwidth utilisation as fewer nodes are involved in rebroadcasting a message. This percentage is calculated as the number of nodes that received the message but did not rebroadcast it divided by the the total number of nodes that received the message. The percentages of saved rebroadcasts as the number of nodes in the network increases from 50 to 250 nodes are illustrated in Figure 4. This figure also shows the influence of parameter control on the percentage of saved rebroadcasts.

The percentage of saved rebroadcasts appears to increase linearly as the number of nodes increases from 50 to 250 nodes for four of the controlled parameters: residual energy (f1), sink distance (f3), node density (f4), and message priority (f5). When analysed in tandem with Figure 3, the percentage of saved rebroadcasts is seen to exhibit the inverse trend compared with that shown for the percentage of rebroadcast nodes and thus shows similar parameter effects. Controlling the sink distance (f3) parameter results in a slight increase in the percentage of saved rebroadcasts, with values ranging from 94% to 99% as the number of nodes increases from 50 to 250. With the activation of the node density (f4) and message priority (f5) parameters, although the percentage of saved rebroadcasts remains relatively stable for networks of 50–150 nodes, it shows significant increases of approximately 7% and 10% as the number of nodes increases to 200 and 250, respectively. The percentage of saved rebroadcasts is shown to be stably increasing with an increasing number of nodes in the network when the residual energy parameter (f5) is controlled. The next-node distance parameter (f2) shows the least stability, as seen from the decrease in the percentage of saved rebroadcasts as the number of nodes increases to 150, followed by a slight increase as the number of nodes in the network grows to 250.

#### 5.2.4. Network Lifetime

**Figure 5:**
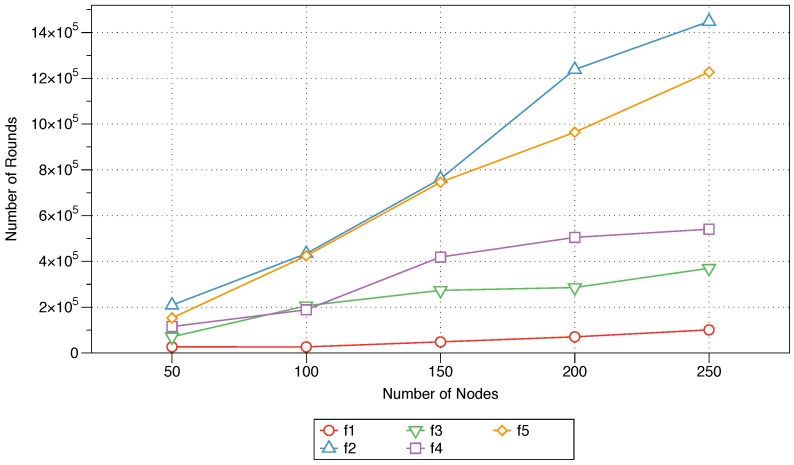
The impact of parameter activation on the average network lifetime as the number of nodes increases from 50 to 250 nodes.

An IoT network or WSN is composed of a variable number of sensors that are deployed in a sensing area. The constrained battery capacity of the sensor nodes is a major factor that impacts the lifetime of such a network [35]. To ensure network continuity, a message should be disseminated evenly among the network nodes. Figure 5 plots the effects of the examined MFWF parameters on the network lifetime, reflecting the ability to ensure even energy dissemination among network nodes as their number increases from 50 to 250. This evaluation metric is represented in the plot as the number of rounds until the death of the last node in the network.

The lifetime of the simulated network increases linearly as the number of nodes increases from 50 to 250 for each of the MFWF parameters. This shows that as the number of nodes in the network increases, the network has a higher chance of staying alive longer because more options for dissemination are presented. The longevity of the network is most evident when the next-node distance parameter (f2) is controlled, where large variations can be seen as the number of nodes grows to 250. This is because a node consumes less energy when sending a message to a neighbouring node that is closer to it, and hence, this prolongs the lifetime of the node and the network. The impacts of the node density (f4) and sink distance (f3) on the network lifetime are similar, with the activation of the node density parameter (f4) outperforming that of the sink distance parameter (f3) as the number of nodes increases to 250. However, this increase is not as evident as that seen with the activation of the next-node distance parameter (f2). The residual energy parameter (f1), by itself, has no effect on the lifetime of the network. By contrast, the activation of the message priority parameter (f5) is seen to be fruitful, as the network lifetime increases with a growing number of nodes in the network.

### 5.3. Summary

The impact of each of the five parameters (energy, next-node distance, sink distance, node density, and message priority) utilised in the proposed MFWF was comparatively assessed to determine their effect on end-to-end delay, rebroadcast nodes, saved rebroadcasts, and network lifetime. That is to support the construction of application-specific MFWFs in Section 6. The end-to-end delay was observed to be the lowest for the sink distance parameter (f3), followed by residual energy (f1) and message priority (f5) (see Figure 2). The sink distance parameter (f3) expectantly produced the best percentage of rebroadcast nodes and saved rebroadcasts (see Figure 3 and Figure 4). Residual energy (f1) and message priority (f5) generated the next best rebroadcast nodes and saved rebroadcasts results. Network lifetime was affected by the next-node distance (f2) with the best longevity of the five parameters (see Figure 5). These findings should inform the construction of the MFWF for various application in the following section (see Section 6). Nonetheless, the parameters were examined in isolation, and therefore other considerations were also taken into account and discussed.

## 6. Effects on Application Requirements

The previous section explored the effects of different parameters on four evaluation metrics: end-to-end delay, rebroadcast nodes, saved rebroadcasts, and network lifetime. The purpose of this investigation is to determine the ideal combination of parameters that can positively impact the most relevant performance measures to satisfy the needs of a given IoT application. However, additional considerations are taken into account since the parameters were studied in silo. In this section, and within the scope of this paper, IoT applications are classified into three main categories:

- Critical IoT applications, which require fast data delivery.
- Energy-efficient IoT applications, which are subject to both power consumption and energy constraints.
- Bandwidth-intensive IoT applications, which require the minimum amount of data to be transmitted over the network.

The rest of this section identifies the most impactful parameters and constructs a corresponding MFWF for each class of IoT applications considered in this paper. This section will also comparatively assess the performance of the constructed MFWFs in terms of the same evaluation metrics described in Section 5 against two benchmarks: the traditional gossiping protocol and FELGossiping.

### 6.1. Critical IoT Applications

Time-critical IoT applications require an ultra-low end-to-end delay to ensure that messages are received as soon as possible. In the motivating scenario (see Section 3), if Mrs. Fatimah’s motion fails to be captured by the motion sensors, Norah (the domiciliary carer) will need to be notified immediately so that she can change her usual route to reach Mrs. Fatimah quickly or notify an ambulance. Based on the results described in Section 5 and summarised in Table 4, to ensure a reduced end-to-end delay for immediate response, the following parameters need to be activated:

- Sink distance (f3): this parameter linearly impacted the performance of the end-to-end as the number of nodes in the network increases. This is demonstrated in Figure 2, where the end-to-end delay for the sink distance parameter proved the lowest of the other five parameters. Therefore, the sink distance (f3) is highly suitable for consideration in critical IoT applications.
- Node density (f4): the number of neighbours is important to ensure successful message delivery. For instance, if a node has no neighbours, then it cannot rebroadcast a message, and hence, the sink node will not receive it. Therefore, the more neighbours there are, the higher the rebroadcast and delivery probabilities.
- Message priority (f5): the message priority parameter is activated, and a critical message is classified as high priority to ensure its rapid delivery. The impact of this parameter was confirmed in Section 5, where the performance of the message priority parameter was the second lowest (after sink distance) as shown in Figure 2.

While the residual energy (f1) when observed in isolation produced low end-to-end delay (see Figure 2), the critical message should be sent to the node that is closest to the sink event if was short on energy. Therefore, the adapted MFWF for critical IoT applications is formulated according to Equation (10):

(10) $$w=f_{3}\cdot p_{3}+f_{4}\cdot p_{4}+f_{5}\cdot p_{5}$$

where $p_{3}$ is defined in Equation (6), $p_{4}$ is defined in Equation (7), and $p_{5}$ is set to 1 for high-priority messages.

### 6.2. Energy-Efficient IoT Applications

The lifetime of an IoT network or WSN depends on the energy-saving techniques that can be adopted to ensure that the constrained sensor nodes are not drained of their energy too quickly. In the case of our motivating scenario (see Section 3), financing the smart domiciliary care system may prove draining to the city, as human life expectancy is on the rise. Therefore, energy-saving strategies to ensure the longevity of the nodes is an attractive prospect for the municipality to ensure the continuity of the IoT system. To support energy-efficiency applications, the following parameters need to be controlled (see Table 4):

- Residual energy (f1): this parameter is activated to ensure that the source node is aware of the next node’s remaining energy to ensure that the message is forwarded to the closest neighbouring node with the highest energy level. While the results in Figure 5 leaves much to be desired for this parameter, to expand the lifetime of the neighbouring nodes their residual energy was considered. This, in turn, will ensure the continuity of the underlying sensor network and the overall energy-efficient application.
- Next-node distance (f2): to reserve a node’s energy, the message is forwarded to the closest neighbouring node. This was validated in Figure 5 as the parameter was observed positively impacting the network lifetime metric as the number of nodes was increased and producing the best longevity results.

Therefore, the MFWF for energy-efficient IoT applications is adjusted based on Equation (11):

(11) $$w=f_{1}\cdot p_{1}+f_{2}\cdot p_{2}$$

where $p_{y1}$ is defined in Equation (2) and $p_{y2}$ is defined in Equation (3).

### 6.3. Bandwidth-Intensive IoT Applications

The smart domiciliary care system considered in this manuscript (see Section 3) offers on-line appointment services that can be utilised by less able residents to ensure timely health checks. When Norah becomes aware of Mr. Ahmed’s need to visit a urologist, she books an on-line appointment with Mr. Ahmed’s general practitioner on the same day to ensure that Mr. Ahmed is referred to the correct specialist by his doctor. This means that there is an enhanced need for bandwidth-saving measures to ensure smooth videoconference communication between Mr. Ahmed and his general practitioner.

The parameters sink distance (f3) proved superior with the lowest percentage of broadcasts and highest percentages of saved rebroadcasts (see Figure 3 and Figure 4). Followed by residual energy (f1) and message priority (f5). These results validate their inclusion in the bandwidth-intensive MFWF. Since the parameters were studied in isolation in Section 5, the performance of bandwidth-intensive MFWF was assessed with and without next-node distance (f2) and node density (f4). The findings showed that the performance of the MFWF was superior with the inclusion of the two parameters f2 and f4. For that purpose, all five parameters are activated in the MFWF to ensure the reduction of the percentage of rebroadcast nodes while also increasing the percentage of saved rebroadcasts, according to Equation (12) (see Table 4):

(12) $$w=f_{1}\cdot p_{1}+f_{2}\cdot p_{2}+f_{3}\cdot p_{3}+f_{4}\cdot p_{4}+f_{5}\cdot p_{5}$$

where $p_{1}$ is defined in Equation (2), $p_{2}$ is defined in Equation (3), $p_{3}$ is defined in Equation (6), $p_{4}$ is defined in Equation (7), and $p_{5}$ is set to 1 for high-priority messages.

### 6.4. Comparative Assessment Results

In this section, the performances of the constructed MFWFs (see Equations (10)–(12)) are comparatively assessed against those of two benchmark protocols, namely, the traditional gossiping protocol and FELGossiping [19] (see Table 1), as the number of nodes in the network increases. The evaluation framework and performance metrics described in Section 5 are also used here; the latter include the end-to-end delay, rebroadcast nodes, saved rebroadcasts, and network lifetime. Each simulation was executed ten times, and the results were averaged and plotted in line graphs, with the number of nodes plotted on the *x*-axis and the performance measure plotted on a linear scale on the *y*-axis. The analysis was conducted separately for each of the four different measures. The detailed analysis for each metric is presented in separate sections, where the performance of the five algorithms is assessed as the size of the WSN network increases from 50 to 250 nodes.

#### 6.4.1. End-to-End Delay

**Figure 6:**
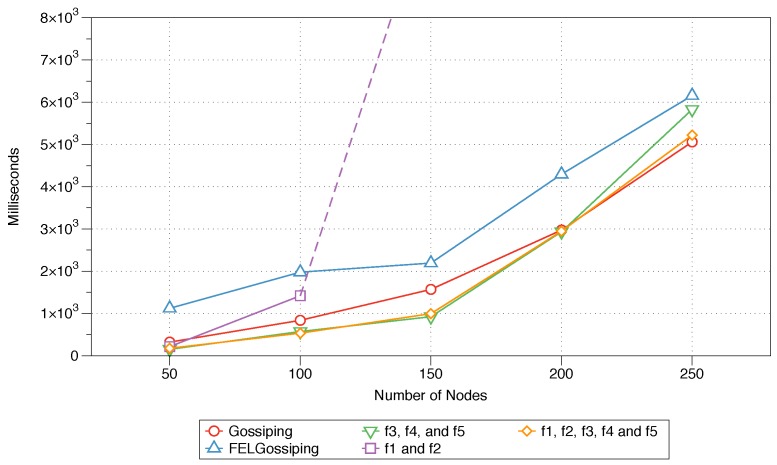
End-to-end delay performances of the three constructed protocols, the traditional gossiping protocol, and FELGossiping with an increasing number of nodes in the network.

The average time differences between the sending and receiving times for all five protocols are shown in Figure 6. The impacts of the five protocols on the evaluation metric as the number of nodes in the network increases from 50 to 250 are displayed. The average end-to-end delay for Equation (11) exceeds the value shown on the vertical axis (presented as a dashed extension in Figure 6, highlighting the difference in performance between this protocol and the other algorithm. This, of course, demonstrates the inadequacy of that equation in reducing delays and, thus, for utilisation for critical IoT applications. The MFWF that is specifically designed for critical IoT application (see Equation (10)), along with Equation (12), outperforms the three other protocols (Equation (11), traditional gossiping, and FELGossiping) as the number of nodes in the network grows. Only slight variations are evident between the performances of the equations for critical and bandwidth-intensive IoT application (Equations (10) and (12), respectively), with the latter performing slightly better as the number of nodes in the network increases to 250. This shows that for a relatively small network (i.e., with fewer than 200 nodes), fewer parameters (f3,f4,f5) need to be accounted for to ensure the minimal delay; however, for a larger network, the remaining parameters should also be considered (f1,f2). The traditional gossiping protocol performs slightly better than FELGossiping, as the additional considerations of FELGossiping (residual energy and hop count to sink) prove detrimental to its end-to-end delay.

#### 6.4.2. Rebroadcast Nodes

**Figure 7:**
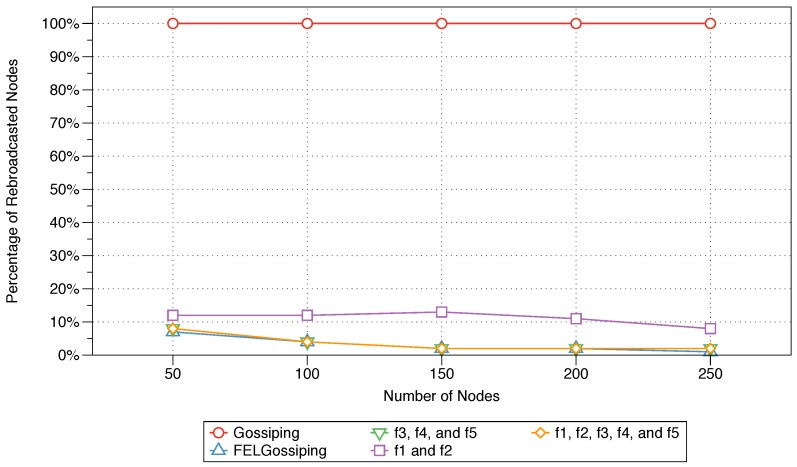
Percentages of rebroadcast nodes for the three constructed protocols, the traditional gossiping protocol, and FELGossiping with an increasing number of nodes in the network.

The percentages of rebroadcast nodes for all three constructed protocols (Equations (10)–(12)), the traditional gossiping protocol, and FELGossiping are shown in Figure 7 for networks with 50 to 250 nodes. The traditional gossiping protocol results in 100% rebroadcast nodes as the number of nodes in the network increases. This means that all nodes in the network receive the message for rebroadcasting, making this protocol ill suited for bandwidth-intensive IoT applications. The percentages of rebroadcast nodes for the MFWFs designed for critical and bandwidth-intensive IoT applications (see Equations (10) and (12), respectively) are comparable, with values ranging from 2% to 8%. These values clearly outperform those of the traditional gossiping protocol. Interestingly, FELGossiping proves to be well suited for routing messages in bandwidth-intensive IoT applications, with a performance comparable to those of the protocols for critical and bandwidth-intensive applications (see Equations (10) and (12), respectively). When only the residual energy (f1) and next-node distance (f2) are considered, as in the MFWF for energy-efficient IoT applications (see Equation (11)), the percentage of rebroadcast nodes slightly increases in comparison to the results for the other constructed protocols (Equations (10) and (12)) and FELGossiping. Nevertheless, the performance of this protocol remains superior to that of the traditional gossiping protocol. For all protocols, the percentage of rebroadcast nodes remains relatively stable as the number of nodes in the network increases, with only slight variations, ranging from none to 4%.

#### 6.4.3. Saved Rebroadcasts

**Figure 8:**
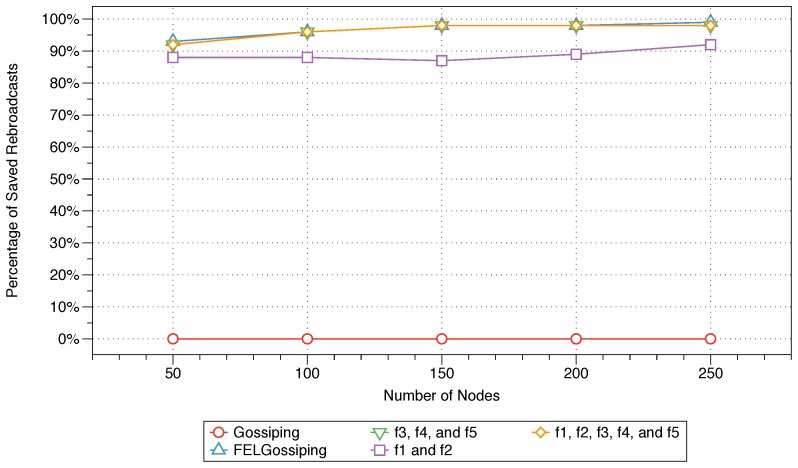
Percentages of saved rebroadcasts for the three constructed protocols, the traditional gossiping protocol, and FELGossiping with an increasing number of nodes in the network.

The performances in terms of percentage of saved rebroadcasts are shown in Figure 8 for all five protocols (Equations (10)–(12), gossiping, and FELGossiping) for networks with 50 to 250 nodes. As anticipated, the performance in terms of this metric shows the inverse behaviour relative to the performance in terms of the percentage of rebroadcast nodes, as shown in Figure 7, because it measures the percentage of bandwidth utilisation. The performance of the MFWFs constructed for critical and bandwidth-intensive IoT applications (see Equations (10) and (12), respectively) prove equally suitable, as the bandwidth utilisation is substantially reduced, with a savings of almost 90% of rebroadcasts. FELGossiping performs comparably well relative to the constructed equations (Equations (10) and (12)), thus confirming this protocol’s adequacy for bandwidth-intensive IoT applications. The MFWF constructed for energy-efficient IoT applications (see Equation (11)) also performs relatively well, considerably reducing the bandwidth utilisation as the number of nodes in the network increases. Similar to what was reported for the percentage of rebroadcast nodes, the bandwidth utilisation of each protocol is relatively stable across different numbers of nodes in the network.

#### 6.4.4. Network Lifetime

**Figure 9:**
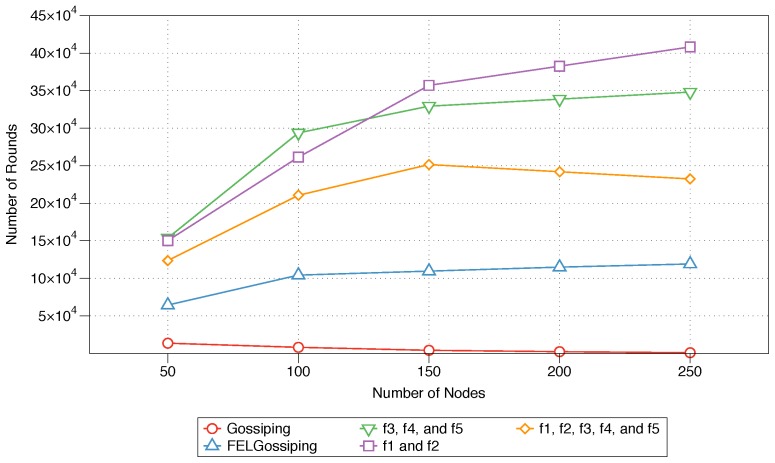
Network lifetime performances of the three constructed protocols, the traditional gossiping protocol, and FELGossiping with an increasing number of nodes in the network.

The average longevity of the network for each of the five protocols is shown in Figure 9. The effect of each assessed protocol is plotted for networks with 50 to 250 nodes. The performance of the MFWF designed for energy-intensive IoT applications (see Equation (11)) is the best as the number of nodes increases to 150, 200, and 250. This, of course, highlights the suitability of this equation for ensuring an increased network lifetime for energy-efficient applications. Interestingly, the performance of the equation developed for critical IoT applications (see Equation (10)) also proves adequate, as the number of rounds until the death of the network is maximised for smaller networks (50 and 100 nodes). Nevertheless, its performance becomes stagnant as the number of nodes in the network increases to 250. FELGossiping causes the network to expire much earlier than the constructed MFWFs do (see Equations (10)–(12)). As expected, the traditional gossiping protocol is the most ill suited for preserving the network lifetime.

### 6.5. Summary

The results of the MFWF’s parameter analysis in Section 5 were used to construct three application-specific MFWFs that were comparatively assessed against the traditional gossiping protocol and FELGossiping [19]. The proposed MFWFs are Equations (10)–(12) for critical, energy-efficient, and bandwidth-intensive IoT applications, respectively. The performance of the proposed protocols was assessed based on the end-to-end delay, rebroadcast nodes, saved rebroadcasts, and network lifetime. The end-to-end delay results show that Equations (10) and (12), which were curated for critical and bandwidth-intensive IoT application, produced the lowest delay; thus, validating their purpose for applications that require a low end-to-end delay, i.e., critical IoT applications (see Figure 6). The percentage of rebroadcasts and saved rebroadcasts’ best performances were when Equations (10) and (12), as well as FELGossiping were utilised (see Figure 7 and Figure 8). Proving their adequacy for bandwidth-intensive IoT application. The findings for the final measure, network lifetime, show that longevity of a network was the longest when the energy-efficient IoT applications’ MFWF (Equation (11)) was used, followed by Equation (10) that was intended for critical applications (see Figure 9). The results of these assessments are summarised in Table 5, where adequate protocols for each class of IoT applications is demonstrated.

## 7. Conclusions and Future Work

This paper proposed a flexible context-aware variant of the gossiping routing protocol for utilisation in WSNs. The new protocol uses an MFWF to enable effective communication among resource-constrained sensors. This MFWF considers several parameters that impact the performance of an IoT network or WSN; these parameters include the residual energy, the distances to the next node and to the sink, the neighbour density, and the message priority. The effects of these parameters on several performance measures were examined to assist in the selection of the most relevant parameters or combinations of parameters for three types of IoT applications (critical, bandwidth-intensive, and energy-efficient). This resulted in three tailored MFWFs, one for each of the application categories (see Equations (10)–(12)).The performances of these three MFWFs were then comparatively assessed against those of the traditional gossiping and FELGossiping [19] protocols and summarised in Table 5. Both critical and bandwidth-intensive MFWFs proved proficient for critical IoT applications, as it took the least delay to reach its destination at varying network scales. For energy-efficient IoT applications, both the energy-efficient MFWF and critical MFWF demonstrated their capability of improving the network lifetime. In the case of bandwidth-intensive IoT applications, three protocols proved equally applicable: critical MFWF, bandwidth-intensive MFWF, and FELGossiping.

Several limitations of the proposed protocol lend themselves to future work. For instance, in this paper, the weighting factors were set only to either 0 or 1; however, considering the possibility of using fractional numbers between 0 and 1 will be valuable. Also, exploring other new parameters and factors to include in the proposed function can make the proposed protocol more adaptive to the requirements of different IoT applications. Additionally, the MFWF parameters were examined in isolation from each other; therefore, the effect of the parameters on each other was not considered experimentally. In the future, this effect will be studied between two or more parameters to better inform the construction of the MFWFs for various IoT applications. Moreover, the overhead caused by message exchanging among nodes will be eliminated by transmitting only neighbouring node’s information that has been changed. The three IoT application discussed in this paper, were considered separately and it would be valuable to study the effect of a mixed traffic IoT and its impact on their performances. Finally, a more thorough investigation of the proposed algorithm will be considered to comparatively assess its performance against other gossip variants and popular IoT protocols.

## Figures and Tables

**Table 1:** A comparison of gossiping protocol variants for WSNs.

Protocol	Year	Parameters	Evaluation Metrics	Network Size
LGossiping [21]	2009	Locations of nodes	Energy consumption	100
			Packet loss	
ELGossiping [22]	2010	Distance to sink	Energy consumption	100
		Residual energy	Hop count	
			Network lifetime	
			Packet loss	
CHGossiping [23]	2010	Distance to sink	Energy consumption	100
		Residual energy	Hop count	
			Network lifetime	
			Packet loss	
FELGossiping [19]	2011	Hop count to sink	Delay	100
		Residual energy	Energy consumption	
			Live nodes	
			Packet loss	
Fuzzy-Gossip [24]	2012	Distance to sink	Energy consumption	100
		Residual energy	Live nodes	
Gossiping-PS [25]	2013	Distance to sink	Energy consumption	100
		Residual energy	Network lifetime	
			Response time	
Dutta et al. [28]	2014	Distance to sink	Energy consumption	100
		Residual energy	Hop count	
			Live nodes	
			Retransmissions saved	
Fitness-Gossip [29]	2015	Distance to neighbour	Delay	100
		Distance to sink	Energy consumption	
		Residual energy	Hop count	
			Live nodes	
DGossip [30]	2016	Distance to sink	Energy consumption	100
		Residual energy	Hop count	
			Live nodes	
			Simulation time	

**Table 2:** Parameter settings for the evaluation environment.

Parameter	Value
Simulation area	80 m × 60 m
Sink node location	(40, 30)
Number of nodes	50, 100, 150, 200, 250
Transmission range	20 m
Initial energy (E)	0.5 joules

**Table 3:** The manipulated factors mapped to the parameters and their associated equations.

Factor	Parameter	Equation
f1	Residual energy	$Energy=\frac{RE}{E}$
f2	Next-node distance	$NeighbourDist=\frac{1}{max(|X-nX|,|Y-nY|)}$
f3	Sink distance	$sinkDist=1-\frac{nToSinkDistance}{sToSinkDistance}$
f4	Node density	$nDensity=\frac{localDensity}{N}$
f5	Message priority	$msg=\left\{\begin{array}{cc} 0 & low\\ 1 & high \end{array}\right.$

**Table 4:** Manipulated parameters for critical, bandwidth-intensive, and energy-efficient applications based on the previous simulation results (see Section 5). The desired impacts on the evaluation metrics for each class of IoT applications considered in this paper are also shown. ↑, −, and ↓ indicate desired increased, average, and reduced impacts, respectively, on an evaluation metric.

Weighted Parameter	IoT Application		
	Critical	Energy-Efficient	Bandwidth-Intensive
(f1) Residual energy		×	×
(f2) Next-node distance		×	×
(f3) Sink distance	×		×
(f4) Node density	×		×
(f5) Message priority	×		×
**Evaluation Metric**				
End-to-end delay	↓	−	−
Rebroadcast nodes	−	−	↓
Saved rebroadcasts	−	−	↑
Network lifetime	−	↑	−

**Table 5:** The suitability of the five protocols (traditional gossiping, FELGossiping, critical MFWF, energy-efficient MFWF, and bandwidth-intensive MFWF) for three categories of IoT applications: critical, bandwidth-intensive, and energy-efficient.

WSN Routing Protocol	IoT Applications		
	Critical	Energy-Efficient	Bandwidth-Intensive
Traditional gossiping			
FELGossiping			✓
Critical MFWF	✓	✓	✓
Energy-efficient MFWF		✓	
Bandwidth-intensive MFWF	✓		✓
